# Surfactant-Laden Janus Droplets with Tunable Morphologies and Enhanced Stability for Fabricating Lens-Shaped Polymeric Microparticles

**DOI:** 10.3390/mi12010029

**Published:** 2020-12-29

**Authors:** Siyuan Xu, Takasi Nisisako

**Affiliations:** 1 Department of Mechanical Engineering, School of Engineering, Tokyo Institute of Technology, Tokyo 152-8550, Japan; xu.s.ab@m.titech.ac.jp; 2 Institute of Innovative Research, Tokyo Institute of Technology, Yokohama 226-8503, Japan

**Keywords:** microfluidics, Janus droplet, surfactant, interfacial tension, stability, microlens

## Abstract

Janus droplets can function as excellent templates for fabricating physically and chemically anisotropic particles. Here, we report new surfactant-laden Janus droplets with curvature controllability and enhanced stability against coalescence, suitable for fabricating shape-anisotropic polymer microparticles. Using a microfluidic flow-focusing device on a glass chip, nanoliter-sized biphasic droplets, comprising an acrylate monomer segment and a silicone-oil (SO) segment containing a surfactant, were produced in a co-flowing aqueous polyvinyl alcohol (PVA) solution. At equilibrium, the droplets formed a Janus geometry based on the minimization of interfacial energy, and each of the two Janus segments were uniform in size with coefficient-of-variation values below 3%. By varying the concentration of the surfactant in the SO phase, the curvature of the interface between the two lobes could be shifted among concave, planar, and convex shapes. In addition, the Janus droplets exhibited significantly improved stability against coalescence compared with previously reported Janus droplets carrying no surfactant that coalesced rapidly. Finally, via off-chip photopolymerization, concave-convex, planar-convex, and biconvex lens-shaped particles were fabricated.

## 1. Introduction

Janus droplets have attracted significant interest recently because of their unique properties originating from their two physically and chemically distinct exposed segments. For example, Janus droplets can be used as templates in the fabrication of various functional microparticles, which are valuable for potential applications as electrically and magnetically driven actuators [1,2], interfacial stabilizers [3] and building blocks for self-assembly [4]. Although conventional methods such as vibration mixing [5] can only produce polydisperse Janus droplets with poorly controlled sizes and compositions, microfluidic technology has recently enabled the production of monodisperse Janus droplets with finely tuned sizes and compositions for a variety of applications [6].

To date, monodisperse Janus droplets comprised of two miscible [7] or two immiscible segments [8] have been produced via droplet microfluidics. While the two miscible segments tend to be mutually mixed over time through convective and diffusive transport, Janus droplets of two immiscible segments can maintain their phase-separated geometry via the minimization of interfacial free energy. For example, monodisperse Janus droplets with mutually immiscible curable and non-curable segments have been produced as templates for fabricating lens-shaped polymer particles for use in microoptics applications [9,10,11]. One approach to produce Janus droplets of two immiscible segments via microfluidics is solvent-evaporation-induced phase separation within a droplet [12]; however, this is a time-consuming process, and a suitable co-solvent is needed. Another approach is to form a two-phase parallel stream by infusing two immiscible droplet phases separately, and subsequently emulsifying it by the coflowing continuous-phase stream, using microfluidic droplet generators such as a T-junction [13], flow-focusing geometry [8,9,10,11], and assembled microcapillaries [3,14].

The morphology of microfluidic Janus droplets comprised of two immiscible compartments can be altered via two major routes. One approach is to change the volume ratio of the two Janus compartments by tuning the flow-rate ratios of the two disperse phases. For example, using this approach, poly(*N*-isopropylacrylamide) Janus hydrogels [15] and crescent-moon-shaped amphiphilic polymer particles [16] with controlled shapes were produced from water-in-oil (W/O) and oil-in-water (O/W) Janus droplets, respectively. Another approach is the tuning of the interfacial tensions because the morphology of a biphasic droplet comprising two immiscible phases surrounded by an external phase is determined by three interfacial tensions among the three phases [17]. For example, the shape of microfluidic Janus droplets comprised of PLGA [poly(lactic-co-glycolic acid)] and silicone oil (SO) was varied by adjusting the concentrations of polyvinyl alcohol (PVA) in the external aqueous phase in the range from 0 to 10 wt.% [18]. Meanwhile, Ge et al. reported the morphological transformation of microfluidic Janus droplets comprised of liquid paraffin and ethoxylated trimethylolpropane triacrylate (ETPTA) between spherical and dumbbell shapes by replacing the ambient solution collected in a petri dish between pure water and a 2.0 wt.% aqueous solution of Pluronic F-127 [19]. Furthermore, the use of stimuli-responsive surfactants has been reported to alternate the morphology of compound droplets between the core-shell and Janus states [20]. To our knowledge, however, control of Janus droplet morphologies simply by adjusting the surfactant concentrations in the dispersed phase has not yet been reported.

Previously, we produced microfluidic Janus droplets comprised of a photocurable acrylate monomer and non-curable SO containing a surfactant in an aqueous sodium dodecyl sulfate (SDS) solution for fabricating biconvex polymer microlenses with tunable imaging properties [9,11]. However, one shortcoming of these droplets is their instability against coalescence after collection, reducing the yield efficiency. Thus, there remains intense demand for new microfluidic Janus droplets with controlled shapes and improved stability against coalescence for fabricating templated lens-shaped particles.

In this paper, we report novel microfluidic Janus droplets with controlled morphology and enhanced stability against coalescence suitable for the fabrication of polymeric microlenses. Using a microfluidic flow-focusing device, we generated biphasic droplets comprised of a photocurable monomer and a SO containing a surfactant in the aqueous PVA solution. Here, we found that the surfactant-laden droplets could form a Janus geometry at equilibrium, unlike previous droplets without the surfactant forming a core-shell geometry [21,22]. The curvature of the interface between the two Janus lobes can be changed by adjusting the concentrations of the surfactant in the SO. In addition, the Janus droplets in the aqueous PVA solution are highly stable against coalescence after off-chip collection, unlike those dispersed in aqueous SDS solution. Finally, bioconvex, planar-convex, and concave-convex polymer microlenses can be synthesized via photopolymerization from Janus droplets carrying surfactants at different concentrations.

## 2. Materials and Methods 

### 2.1. Microfluidic Device

A microfluidic flow-focusing droplet generator with a Y-shaped channel and a deeper drainage channel was prepared on a glass chip (15 × 15 × 3.5 mm^3^) using our previously published protocol [22]. The microchannels are rectangular in cross-section. The Y-shaped channel is used for two organic phases, with two co-flowing channels for aqueous streams of 100 μm deep × 100 μm wide around the sheath-focusing junction; the drainage channel is 200 μm deep × 200 μm wide.

### 2.2. Chemicals

Ethanol (>99.5%), acetone (>99%), SDS were purchased from FUJIFILM Wako Pure Chemical Corporation (Osaka, Japan). The acrylate monomer (1,6-hexanediol diacrylate (HDDA), dynamic viscosity *η*_m_ = 6.35 mPa s, density *ρ*_m_ = 1.02 g cm^−3^, Shin-Nakamura Kagaku, Wakayama, Japan) was chosen as the curable droplet phase. A photoinitiator (Darocur 1173, BASF Japan, Tokyo, Japan) was dissolved in HDDA to prepare a 2.0 wt.% mixture for photo-induced crosslinking. For a non-curable droplet phase, a surfactant [cyclopentasiloxane and polyoxyethylene glycol/polyoxypropylene glycol-19/19 dimethicone (BY11-030), HLB 3.5, Dow Corning Toray, Tokyo, Japan] was dissolved in 10-cSt SO (SH200-10CS, Dow Corning Toray) to prepare mixture solutions with concentrations ranging from 0.1 to 5.0 wt.%. For the continuous aqueous phase to produce the surfactant-laden Janus droplets, polyvinyl alcohol (GL-03, *M*_w_~20,000 g mol^−1^, 87%–89% hydrolyzed, Mitsubishi Chemical Corporation, Tokyo, Japan) was dissolved in deionized water (Merck Direct-Q UV, Tokyo, Japan) to prepare a 2.0 wt.% solution. For the continuous aqueous phase to produce surfactant-free Janus droplets, SDS was dissolved in deionized water to prepare a 0.3 wt.% solution. Oil-soluble dye (Oil red O, Sigma-Aldrich, St. Louis, MO, USA) was dissolved in the HDDA solution to visually differentiate the two organic phases.

### 2.3. Preparation of Droplets and Lens-Shaped Polymer Particles

Three syringe pumps (KDS200, KD Scientific, Holliston, MA, USA) and glass syringes (1000 series, Hamilton Company, Reno, NV, USA) were used to infuse the fluids into the microfluidic device to generate droplets. The curable segments of the generated Janus droplets were solidified continuously via off-chip photopolymerization using an ultraviolet (UV) light source (LA-410UV, Hayashi-repic, Tokyo, Japan) with an irradiation distance of approximately 15–20 cm. The products were filtered using a nylon mesh sheet (grid size: 42 × 42 μm, Tokyo Screen, Tokyo, Japan) to remove smaller particles produced from satellite droplets. The particles were lightly washed using acetone and ethanol to remove residual non-curable fluid and dust.

### 2.4. Characterization

Droplet formation in the microfluidic device was monitored using an optical microscope (BX-51, Olympus, Tokyo, Japan) equipped with a high-speed video camera (Fastcam Mini AX50, Photron, Tokyo, Japan). The interfacial tensions between the two liquids were measured using the pendant-drop method (B100, Asumi Giken, Tokyo, Japan). We used the software ImageJ (National Institutes of Health, New York, NY, USA) to measure the aperture diameters of Janus droplets. We also used a scanning electron microscope (SEM, JSM-6610LA, JEOL, Tokyo, Japan) to observe the shape of the polymer particles. Surface Evolver [23] was used to simulate the effect of interfacial tension on the shape of droplets and particles.

## 3. Results and Discussion

### 3.1. Generation of Biphasic Janus Droplets

**Figure 1:**
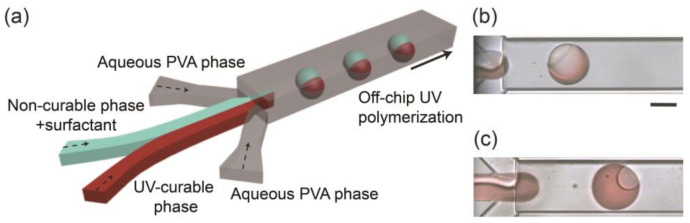
Microfluidic formation of biphasic Janus droplets. (**a**) Schematic illustration of a microfluidic Janus droplet generator with a deeper drainage region. (**b**,**c**) Formation of Janus droplets when flow rate ratios of acrylate monomer (*Q*_m_) and silicone oil (*Q*_s_) are (**b**) *Q*_m_:*Q*_s_ = 1:1 and (**c**) *Q*_m_:*Q*_s_ = 11:1. Total flow rate of the two droplet phases (*Q*_d, total_ = *Q*_m_ + *Q*_s_) is 0.6 mL h^−1^, and the flow rate of the aqueous polyvinyl alcohol (PVA) phase (*Q*_c_) is 8.0 mL h^−1^ (4.0 mL h^−1^ × 2). Scale bar: 100 μm.

Monodisperse biphasic droplets were produced at the sheath-focusing junction one by one when we infused HDDA and SO containing the surfactant as the curable and non-curable disperse phases and the aqueous PVA solution as the continuous phase at appropriate flow rates (Figure 1a). For example, when the flow rates of HDDA (*Q*_m_), SO (*Q*_s_), and the PVA solution (*Q*_c_) were 0.3, 0.3, and 8.0 mL h^−1^, respectively, the production rate was ~78 drops s^−1^ (Figure 1b). Compared with the droplets immediately following their break-off, the Janus morphology could be observed more clearly when they were flowing through the drainage tube (see Supplementary Figure S1a), suggesting interfacial-energy-driven structural evolution. By modulating the flow rates at low Reynolds and capillary numbers where droplets could form regularly [7,8], the droplet size and production rate could be varied in the range of 101–242 μm and 18–205 drops s^−1^, respectively (Figure S2). The volume ratios of the two Janus compartments could be easily varied by changing the flow rate ratio of the two disperse phases (Figure 1c and Figure S1b–e).

**Figure 2:**
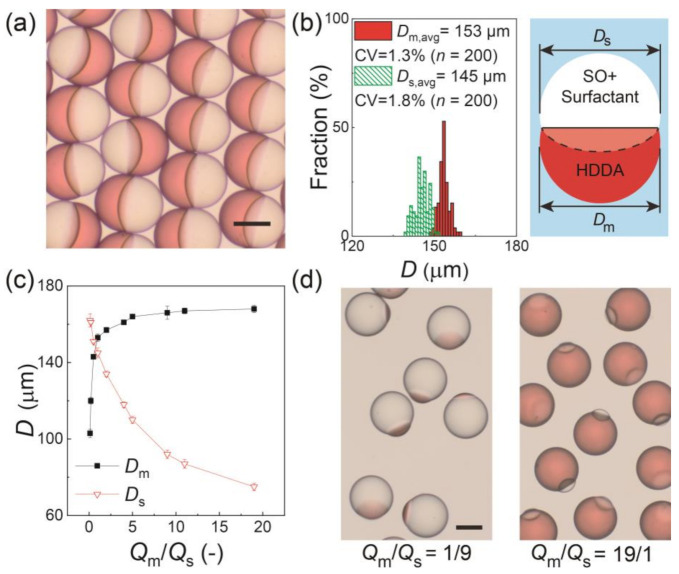
Characterization of Janus droplets with surfactant concentration of 0.1 wt.% at equilibrium. (**a**) Photomicrograph of Janus droplets generated at *Q*_m_:*Q*_s_ = 1:1. (**b**) Size distributions of Janus segments in (**a**). The inset image presents the measured dimensions *D*_m_ and *D*_s_. (**c**) Experimental relationship between *Q*_m_/*Q*_s_ and droplet size. The error bars indicate standard deviation of the measured droplet sizes (*n* = 200) (**d**) Photomicrographs of Janus droplets generated at *Q*_m_:*Q*_s_ = 1:9 (left) and 19:1 (right) when *Q*_d, total_ = 0.6 mL h^−1^ and *Q*_c_ = 8.0 mL h^−1^. Scale bars: 100 μm.

Figure 2a shows the surfactant-laden Janus droplets produced at *Q*_m_/*Q*_s_ = 1/1 and collected on a glass slide. All the droplets had a snowman-like Janus geometry with a concave-convex HDDA segment and biconvex SO segment separated by a curved inner interface. The aperture diameters of the HDDA (*D*_m_) and SO (*D*_s_) segments were measured, and they had narrow size distributions with coefficient-of-variation (CV) values of approximately 2% (Figure 2b), indicating that the droplets were highly monodisperse. The aperture diameters could be controlled by changing the *Q*_m_/*Q*_s_. As *Q*_m_/*Q*_s_ increased from 1/9 to 19/1, the sum of *Q*_m_ and *Q*_s_ remained constant (0.6 mL h^−1^), *D*_m_ increased from 103 to 168 μm with the CV in the range of 1.0% to 2.2%, and *D*_s_ decreased from 162 to 75 μm with the CV in the range from 0.8% to 2.4% (Figure 2c,d). 

Previously, we reported that biphasic droplets comprised of HDDA and SO without surfactant formed a core-shell geometry at equilibrium in aqueous PVA solution even if their initial morphology was Janus [21,22]. In contrast, in this study, we found that the simple addition of the surfactant to the SO phase resulted in the Janus morphology of the droplets at equilibrium. This difference in morphology can be explained by the three spreading parameters *S_i_* = *γ_jk_* − (*γ_ij_* + *γ_ki_*) [17], where, *γ_jk_*, *γ_jk_*_,_ and *γ_jk_* are the three interfacial tensions at the three interfaces (*i* ≠ *j* ≠ *k* = 1, 2, 3). In this study, indices 1, 2, and 3 were for the SO, external aqueous phase, and HDDA, respectively. When indices 1 and 3 for the two phases of droplets satisfy *γ*_12_ > *γ*_23_, *S*_1_ remains negative and the biphasic compound droplets dispersed in the external phase exhibit three morphologies at equilibrium: (1) *S*_1_ < 0, *S*_2_ < 0, *S*_3_ > 0: core-shell droplets (or fully engulfed), where phase 1 is completed engulfed by phase 2; (2) *S*_1_ < 0, *S*_2_ < 0, *S*_3_ < 0: Janus droplets (or partially engulfed); and (3) *S*_1_ < 0, *S*_2_ > 0, *S*_3_ < 0: two separate drops. 

To illustrate this point, we measured the three interfacial tensions and calculated the three spreading parameters. In our previous study without a surfactant, we obtained *γ*_31_ = 2.2 mN m^−1^, *γ*_12_ = 10.8 mN m^−1^, and *γ*_23_ = 1.1 mN m^−1^, thereby causing *S*_1_ < 0, *S*_2_ < 0, *S*_3_ > 0, forming a core-shell structure [21]. Meanwhile, in this study, when we used the SO containing the surfactant at 0.1 wt.%, the average values and standard deviation of the measured interfacial tensions were *γ*_31_ = 1.63 ± 0.07 mN m^−1^ and *γ*_12_ = 9.61 ± 0.06 mN m^−1^, while *γ*_23_ maintained a similar value of 1.13 ± 0.02 mN m^−1^ (*n* = 5, Figure S3). These values correspond to the core-shell condition (*S*_1_ < 0, *S*_2_ < 0, *S*_3_ > 0), which is inconsistent with the Janus morphology we confirmed in this study. One possible reason for this discrepancy is that there might be some HDDA molecules at the interface between the SO and the aqueous PVA phase, reducing the interfacial energy at the SO/water interface [8]. This reduced interfacial tension (*γ*′_12_) was estimated using the following equation [24]:

(1) $$\gamma_{12}^{\prime }=\gamma_{23}\cos\alpha +\gamma_{31}\cos\beta$$

where *α* and *β* are the contact angles, as shown in Figure S4. We found that the average values and standard deviation of the measured contact angles were *α* = 10.4 ± 1.0° and *β* = 35.6 ± 0.6° (*n* = 5) in this case, and we obtained *γ*′_12_ ~2.17 mN m^−1^. This satisfies *S*_1_ < 0, *S*_2_ < 0, *S*_3_ < 0, and the condition for a Janus morphology.

### 3.2. Effect of Inner Surfactant Concentration on Janus Morphology

**Figure 3:**
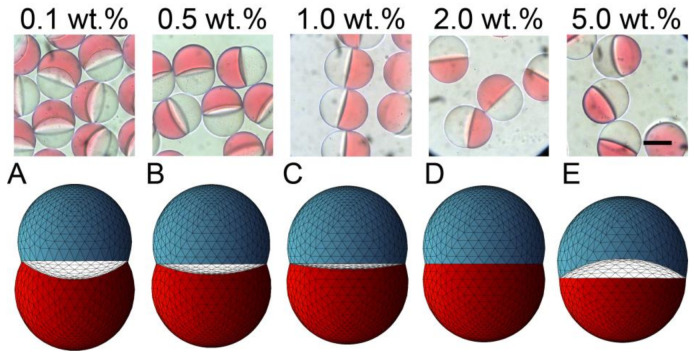
Effect of surfactant concentration on the morphology of Janus droplets. Photomicrographs showing structural variation of Janus droplets carrying surfactant at concentrations ranging from 0.1 to 5.0 wt.% (top) and their corresponding simulated three-dimensional shapes ((**A**–**E**), bottom). Flow conditions were *Q*_m_ = *Q*_s_ = 0.3 mL h^−1^, and *Q*_c_ = 8.0 mL h^−1^. Scale bar: 100 μm.

**Figure 4:**
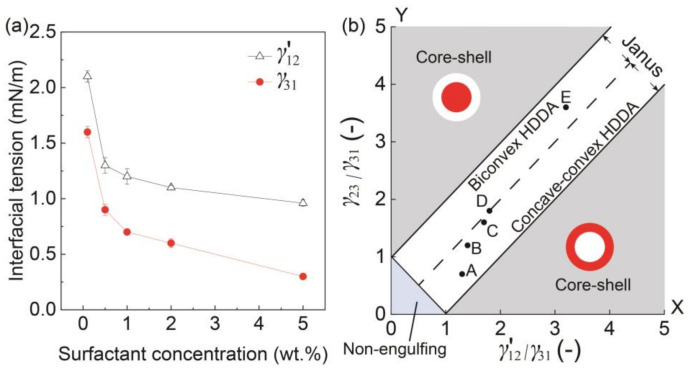
Morphology in response to interfacial tension. (**a**) Surfactant concentrations in silicone vs. interfacial tensions *γ*′_12_ and *γ*_31_. (**b**) Phase diagram showing the relation between the ratio of interfacial tensions *γ*′_12_/*γ*_31_ and the morphologies of the biphasic droplets when *Q*_m_/*Q*_s_ = 1. Inset points of A to E correspond to the results in Figure 3.

We tested the SO phases with different surfactant concentrations to verify its effect on the morphology of the produced biphasic droplets (Figure 3). The free software Surface Evolver [23] was used to estimate the three-dimensional (3D) morphology of the produced droplets based on the measured/calculated interfacial tensions *γ*_31_, *γ*_23,_ and *γ*′_12_ (Figure 4a).

Different Janus morphologies were observed when we varied the concentration of the surfactant in the SO phase in the range from 0.1 to 5.0 wt.% under the same flow condition (Figure 3 and Figure S1b–e). Typically, the increase of surfactant concentration in the SO phase led to the curvature variation of the inner HDDA/SO interface, causing a variation in the shape of the HDDA segment from concave-convex to biconvex, and that of the SO segment from biconvex to concave-convex. When the surfactant concentration in the SO phase was low (0.1, 0.5, or 1.0 wt.%), the HDDA and SO segments of the droplets produced at *Q*_m_/*Q*_s_ = 1 were in concave-convex and biconvex shapes, respectively (Figure 3A,C). At 2.0 wt.%, the two segments became hemispherical with the planar interface (Figure 3D). At 5.0 wt.%, biconvex HDDA and concave-convex SO segments were obtained (Figure 3E). These experimental results agree well with the modeled shapes. 

A similar variation in the curvature of the inner HDDA/SO interface could also be confirmed as the surfactant concentration was varied at different flow rate ratios of the two dispersed phases (*Q*_m_/*Q*_s_ of 1/2 and 2/1, Figure S5), although the ranges of the concentrations for the concave-convex and biconvex shapes were different. The HDDA and SO segments produced at *Q*_m_/*Q*_s_ = 2/1 were concave-convex and biconvex, respectively, for surfactant concentrations from 0.1 to 2.0 wt.%; at 5.0 wt.%, the HDDA and SO segments became biconvex and concave-convex, respectively (Figure S5a). Meanwhile, the droplets produced at *Q*_m_/*Q*_s_ = 1/2 had concave-convex HDDA and biconvex SO segments for values of <1.0 wt.% and biconvex HDDA and concave-convex SO segments for values of >1.0 wt.% (Figure S5b).

To better understand the effect of surfactant concentration on the variation of Janus configurations, we plotted the conditions under different surfactant concentrations (Figure 3A–E) on a phase diagram that can theoretically estimate the possible morphologies of a biphasic droplet (i.e., core-shell, Janus, and non-engulfing states, Figure 4b) based on the interfacial tensions [9,25]. The white region in the middle of the diagram shows a Janus morphology, where the biconvex HDDA and concave-convex SO segments form above the *Y* = *X* line, and the concave-convex HDDA and biconvex SO segments form below the *Y* = *X* line. The HDDA/SO interface becomes planar if the condition is on the line of *Y* = *X*. As shown in the phase diagram, all the experimental conditions (A–E) plot in the Janus region, with A, B, and C are in the region for the concave-convex HDDA segment, D nearly on the line of *Y* = *X* for the planar HDDA/SO interface, and E in the biconvex HDDA region. These results are in good agreement with the experimental results (shown in Figure 3). Thus, we can visualize how droplet morphology changes when we increase/decrease the surfactant concentration.

### 3.3. Stability Assessment of Janus Droplets

The stability of the produced Janus droplets against coalescence is important for their application, including the fabrication of polymeric microlenses at high yields. Therefore, by using optical microscopy, we compared the off-chip stability of surfactant-laden Janus droplets in an aqueous PVA solution with surfactant-free Janus droplets in an aqueous SDS solution [8,13] at room temperature (23 °C). 

**Figure 5:**
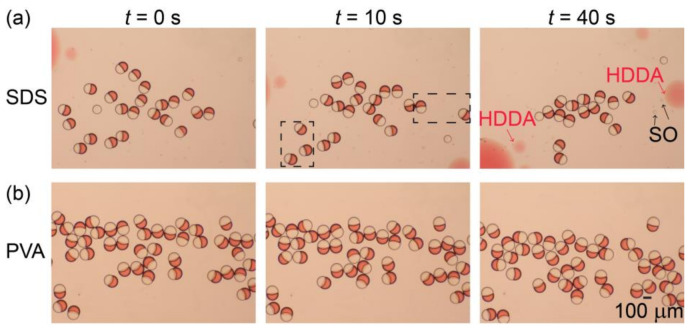
Off-chip stability of Janus droplets. (**a,b**) Comparison of off-chip stability between (**a**) surfactant-free Janus droplets dispersed in aqueous sodium dodecyl sulfate (SDS) solution and (**b**) Janus droplets containing 0.1 wt.% surfactant in their silicone-oil segments dispersed in aqueous PVA solution. Both droplets were produced at *Q*_m_ = *Q*_s_ = 0.3 mL h^−1^, and *Q*_c_ = 8.0 mL h^−1^.

Figure 5a illustrates the stability of the surfactant-free Janus droplets in the 0.3 wt.% aqueous SDS solution produced at *Q*_m_/*Q*_s_ = 1/1 (for their formation in the device, see Figure S1f). After collection in a petri dish (at *t* = 0), the droplets floated at the air-water interface, maintaining their Janus morphology for a short period of time (*t* = 10 s). However, at *t* = 40 s, we found that many Janus droplets were already lost, suggesting their rupturing due to coalescence. In contrast, for the surfactant-laden Janus droplets in the aqueous PVA solution, no coalescence was observed at *t* = 40 s, suggesting better stability (Figure 5b). Similar tendencies were obtained for both surfactant-free and surfactant-laden Janus droplets produced at *Q*_m_/*Q*_s_ = 2/1 and 1/2 (Figure S6).

**Figure 6:**
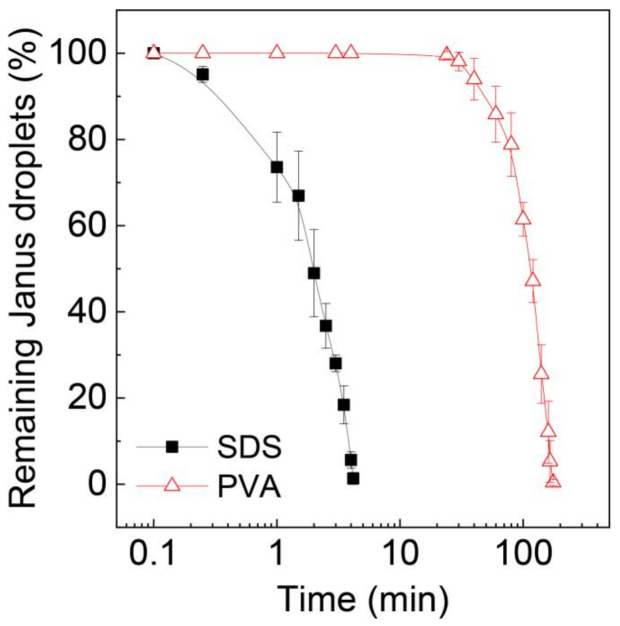
Decrease in Janus droplets by coalescence over time. Surfactant concentration in the silicone oil phase of the Janus droplets in aqueous PVA solution is 0.1 wt.%. Droplets were produced at *Q*_m_ = *Q*_s_ = 0.3 mL h^−1^, and *Q*_c_ = 8.0 mL h^−1^. The error bars indicate standard deviation of the measurements (*n* = 3).

To further validate the stability of these Janus droplets, we extended the time of observation and counted the number of remaining Janus droplets over time, as shown in Figure 6. The number of surfactant-free Janus droplets in the SDS solution rapidly decreased over time, and all of them ruptured within ~4 min. In contrast, for the surfactant-laden droplets, no coalescence was observed for ~24 min, and it took ~176 min for all the droplets to lose their Janus morphology. 

This significantly improved stability of the surfactant-laden Janus droplets might be due to the molecular size of PVA, which is much larger than the SDS molecules, thereby causing steric hindrance to stabilize the droplets [26]. This could also be attributable to the reduction of the interfacial tensions at the interface between the dispersed and continuous phases, as reported in the generation of Janus [27] and general emulsions [28,29]. For the Janus droplets in the aqueous SDS phase, the interfacial tensions were *γ*_23_ = 4.5 mN m^−1^ and *γ*′_12_ = 5.3 mN m^−1^ [8,13]. Meanwhile, as described above, the SO containing the surfactant at 0.1 wt.% and the aqueous PVA solution gave rise to significantly decreased interfacial tension values (*γ*_23_ = 1.1 mN m^−1^ and *γ*′_12_ = 2.1 mN m^−1^). Such a reduction in interfacial tension might also be responsible for the improved stability against coalescence. A similar phenomenon was reported in the literature, where the presence of phosphatidylcholine as a surfactant in the oil phase at a concentration of 3.0 wt.% reduced the interfacial tension at the oil/water interface to 0.5 mN m^−1^, resulting in more stable O/W Janus droplets with greater resistance to coalescence [30]. 

### 3.4. Fabrication and Characterization of Microlens-Shaped Particles

**Figure 7:**
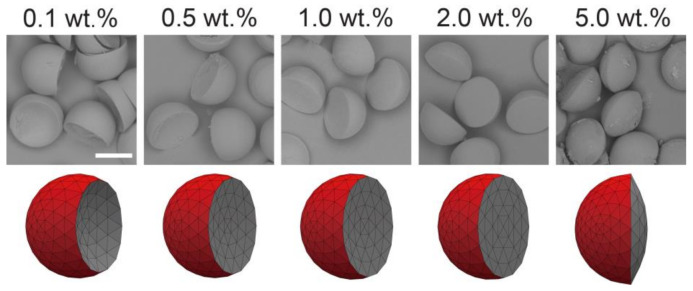
Scanning electron microscopy images of concave-convex, planar-convex, and biconvex particles (**top**), and their simulated models (**bottom**) prepared at different surfactant concentrations in silicone oil segments. Precursor Janus droplets were produced at *Q*_m_ = *Q*_s_ = 0.3 mL h^−1^, and *Q*_c_ = 8.0 mL h^−1^. Scale bar: 100 μm.

Polymer particles of various lens shapes were prepared from the surfactant-laden Janus droplets shown in Figure 3 via off-chip photopolymerization (Figure 7). We confirmed that the fabricated particles reflected the shape of the curable HDDA segment in the precursor Janus droplets, which were concave-convex, planar-convex, and biconvex shapes based on the concentration of the surfactant in the SO. From the precursor droplets carrying the surfactant at lower concentrations (i.e., 0.1, 0.5, and 1.0 wt.%), concave-convex particles with different curvature radii of the concave surface were obtained; more concave surfaces were observed for the particles with lower surfactant concentrations. Surfactant concentrations of 2.0 and 5.0 wt.% yielded planar-convex (i.e., hemispherical) and biconvex particles, respectively. We confirmed similar shape variation from concave-convex to biconvex when the droplets produced at *Q*_m_/*Q*_s_ = 1/2 and 2/1 were solidified, although the transition of the inner curvature between concave and convex surfaces was at 1.0 wt.% for *Q*_m_/*Q*_s_ = 1/2 and 5.0 wt.% for *Q*_m_/*Q*_s_ = 2/1. All the shapes of the particles agreed well with the shape of the HDDA segments in the precursor droplets as well as the results predicted by modeling (Figure S7).

Polymer particles of various microlens shapes can be prepared from similar surfactant-free [8,13] or surfactant-laden [9,11] microfluidic Janus droplets dispersed in an aqueous SDS solution, as demonstrated previously. Meanwhile, we, for the first time, found that surfactant-laden Janus droplets could form in aqueous PVA solution at equilibrium. We believe that the surfactant-laden Janus droplets in this study are more suitable for fabricating templated particles because of their significantly improved stability against coalescence and resultant high yielding efficiency. In addition, for the first time, we revealed that the Janus morphology and resultant particle shapes could be altered simply by changing the surfactant concentrations in the non-curable phase, in addition to tuning the flow-rate ratios of the two disperse phases.

The presented approach could be further enhanced for the fabrication of more shape-anisotropic particles through the variation of the surfactant types, their concentrations, and the number of inputs for the curable or non-curable phases [10]. Moreover, the properties of particles can be extended through a combination of selectively absorbed nanoparticles [31] or magnetic nanoparticles [32]. For industrial-scale production of these droplets and particles, parallelization of many devices on a single substrate [33] will be useful. In addition to the presented particles, the combination of the liquids and surfactants demonstrated in this study might also be applicable for the synthesis of anisotropic polymer microfibers [34].

## 4. Conclusions

In this study, we synthesized surfactant-laden Janus droplets comprised of two immiscible curable and non-curable segments with high stability against coalescence and tunable morphologies, suitable for the fabrication of lens-shaped microparticles. Monodisperse biphasic droplets of an acrylate monomer and SO containing surfactant were produced in an aqueous PVA solution via microfluidic flow focusing. Unlike similar biphasic droplets without surfactants that form a core-shell geometry, we found that the produced droplets had a uniform Janus geometry at equilibrium. Furthermore, the curvature radii of the interface between the two Janus lobes could be varied by varying the concentration of the loaded surfactant. In addition, the produced surfactant-laden Janus droplets had considerably higher stability against coalescence than the surfactant-free Janus droplets in the aqueous SDS solution. We believe that the presented approach provides a new route for fabricating novel particulate materials with precisely designed curvatures, which hold considerable potential as elements in micro optical applications.

## Supplementary Materials

The following are available online at https://www.mdpi.com/2072-666X/12/1/29/s1, Figure S1: Janus droplet formation at different surfactant concentrations and flow-rate ratios, Figure S2: A flow pattern diagram and the effect of the continuous phase flow rate, Figure S3: Measurement of interfacial tensions, Figure S4: Balance of the three interfacial tensions, Figure S5: Janus droplets carrying 0.1 to 5.0 wt.% surfactant, produced at *Q*_m_/*Q*_s_ = 2/1 and 1/2, Figure S6: Off-chip stability of Janus droplets produced at *Q*_m_/*Q*_s_ = 2/1 and 1/2, Figure S7: Lens-shaped polymer particles produced at *Q*_m_/*Q*_s_ = 2/1 and 1/2.
