# Supplementary material for: Surfactant-Laden Janus Droplets with Tunable Morphologies and Enhanced Stability for Fabricating Lens-Shaped Polymeric Microparticles
Source: Micromachines (Basel). 2020 Dec 29;12(1):29. doi: 10.3390/mi12010029 (PMC7824708; doi:10.3390/mi12010029)
Supplement: Supplementary file 1 [file micromachines-12-00029-s001.pdf]

Supplementary Material

Surfactant-laden Janus droplets with tunable morphologies and enhanced stability for fabricating lens-shaped polymeric microparticles

Siyuan Xu and Takasi Nisisako

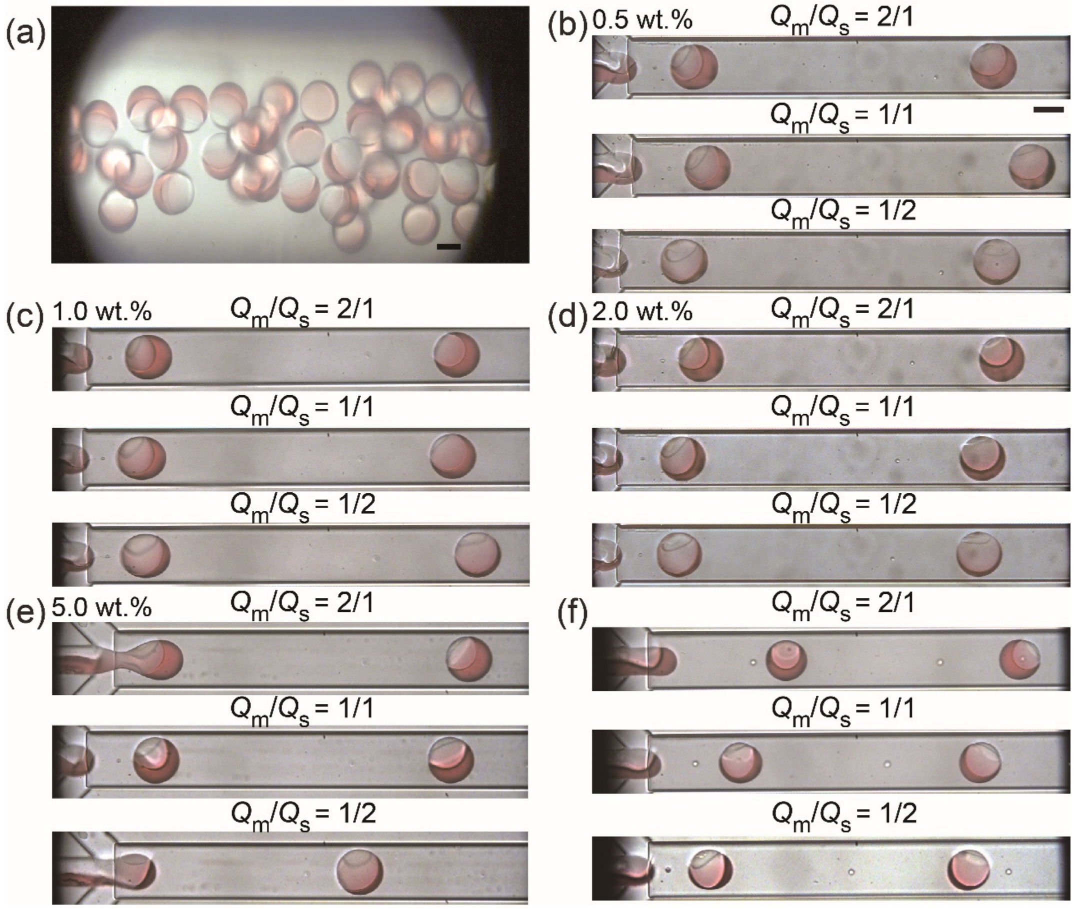

Figure S1. Janus droplet formation at different surfactant concentrations and flow-rate ratios. (a) Biphasic droplets in Fig.1b flowing through the drain tube. (b-e) Janus droplets in the aqueous PVA solution with the concentration of surfactant in the silicone-oil segment at (b) 0.5 wt.%, (c) 1.0 wt.%, (d)2.0 wt.%, and (e) 5.0 wt.%, produced with Q_m/Q_s at 2/1, 1/1 and 1/2. (f) Formation of surfactant-free Janus droplets in the aqueous SDS solution with Q_m/Q_s at 2/1, 1/1 and 1/2. $Q_{d,\ total}$ = 0.6 mL h^{-1}, Q_c = 8.0 mL h^{-1}. Scale bars: 100 µm.

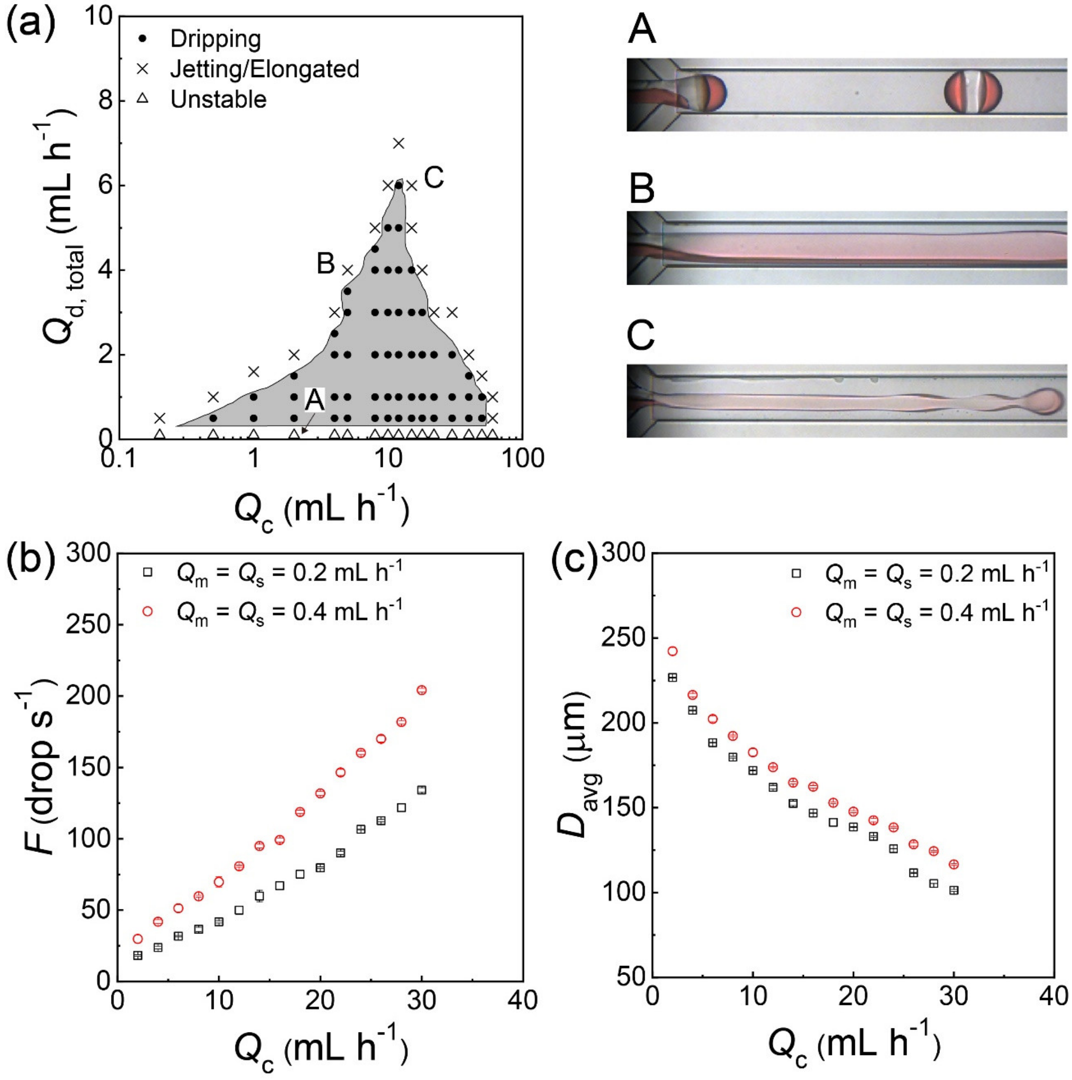

Figure S2. (a) A flow pattern diagram showing the condition for the formation of Janus droplets with the surfactant concentration of 0.1 wt.%. The open triangles represent the condition where the two-phase parallel stream of acrylate monomer and silicone oil (SO) becomes unstable (inset A). The crosses represent the condition of elongated steam (inset B) or irregular jetting regime (inset C). (b) The continuous phase flow rate Q_c vs. generation frequency F. (c) The Q_c vs. average diameter of the biphasic droplets D_{avg}, calculated from F and $Q_{d,\ total}$, based on the assumption that droplets are spherical.

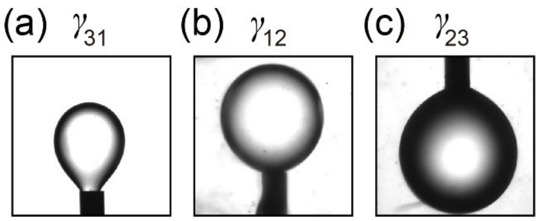

Figure S3. Measurements of interfacial tensions by the pendant-drop method. (a,b) A droplet of SO containing the surfactant at 0.1 wt.% surrounded by (a) HDDA, and (b) aqueous PVA solution. (c) A droplet of HDDA surrounded by the aqueous PVA solution. All measurements were performed by injecting a 10-μL droplet.

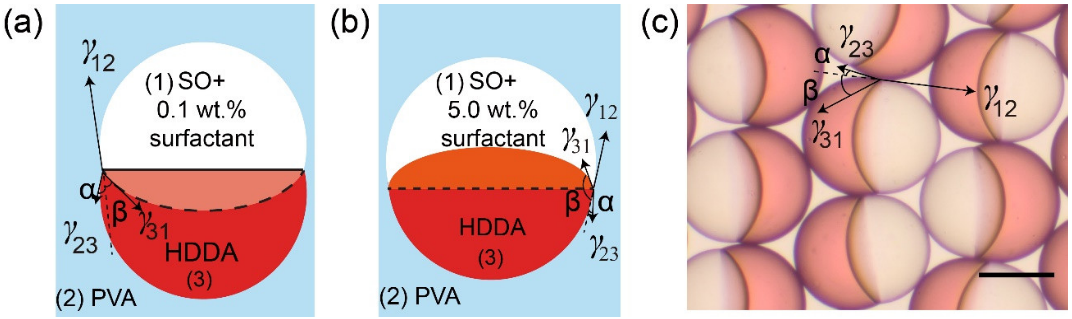

.

Figure S4. (a-b) Schematic illustration of Janus droplets with the surfactant concentration in the SO segment of (a) 0.1 wt.% and (b) 5.0 wt.%. (c) A bright-field image showing the contact angles of α and β in (a). Q_m:Q_s = 1:1. Scale bar: 100 μm.

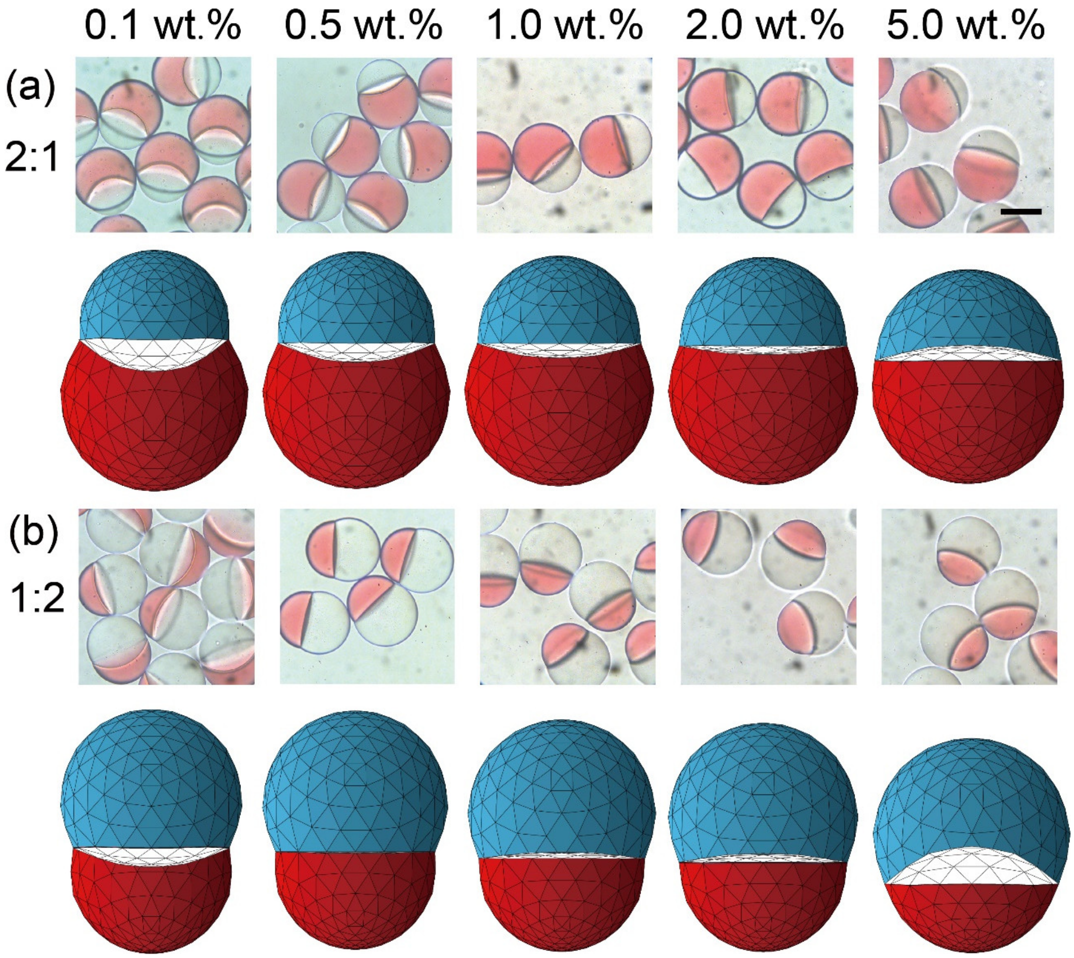

Figure S5. Morphology variation of Janus droplets with the concentration of surfactant in the SO segment ranging from 0.1 to 5.0 wt.% (top) and their corresponding 3D shapes (bottom) when (a) Q_m:Q_s = 2:1, and (b) Q_m:Q_s = 1:2. Droplets were produced at $Q_{d,\ total}$ = 0.6 mL h^{-1}, and Q_c = 8.0 mL h^{-1}. Scale bar: 100 μm.

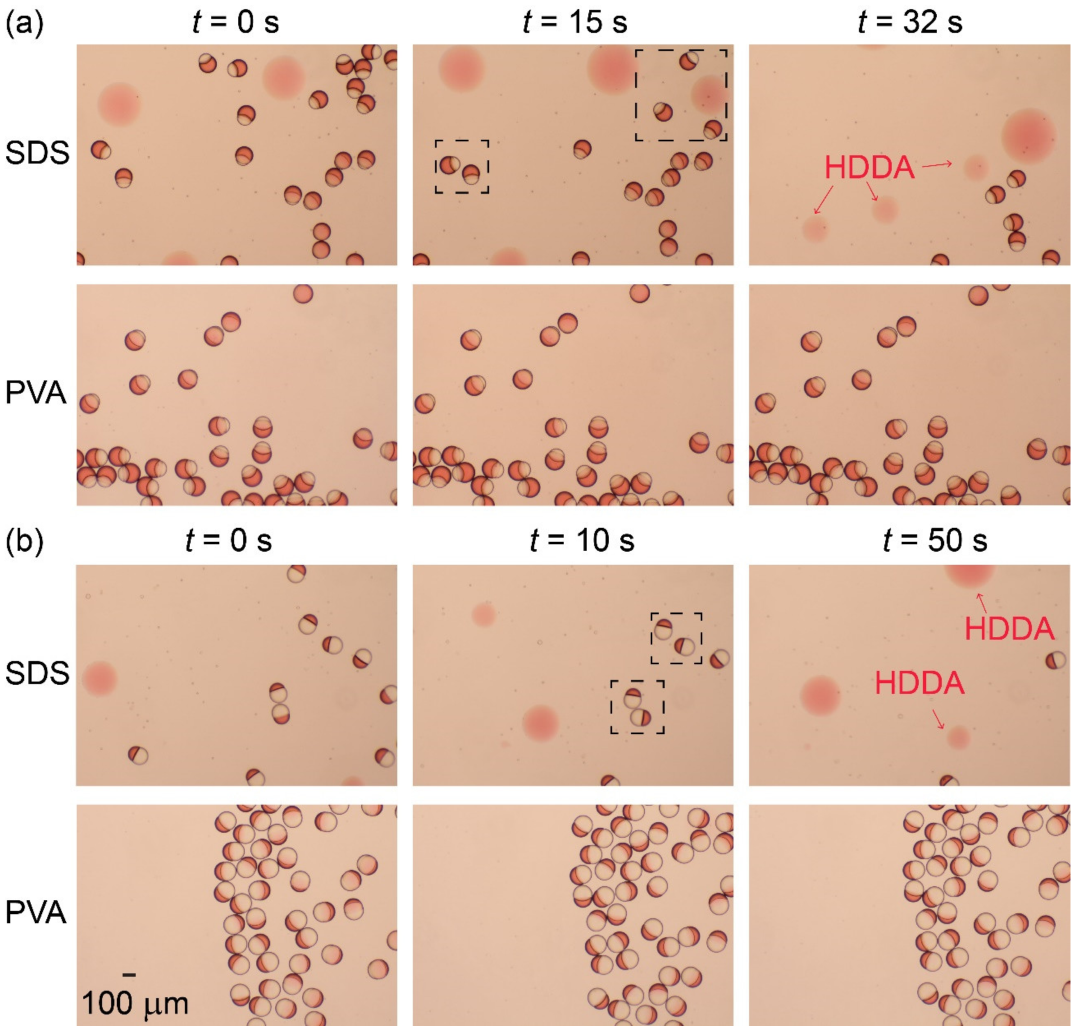

Figure S6. Comparison of off-chip stability between surfactant-free Janus droplets floating in the aqueous SDS phase (top) and Janus droplets containing 0.1 wt.% surfactant floating in aqueous PVA phase (bottom) when (a) Q_m:Q_s = 2:1, and (b) Q_m:Q_s = 1:2. Droplets were produced at $Q_{d,\ total}$ = 0.6 mL h^{-1}, and Q_c = 8.0 mL h^{-1}.

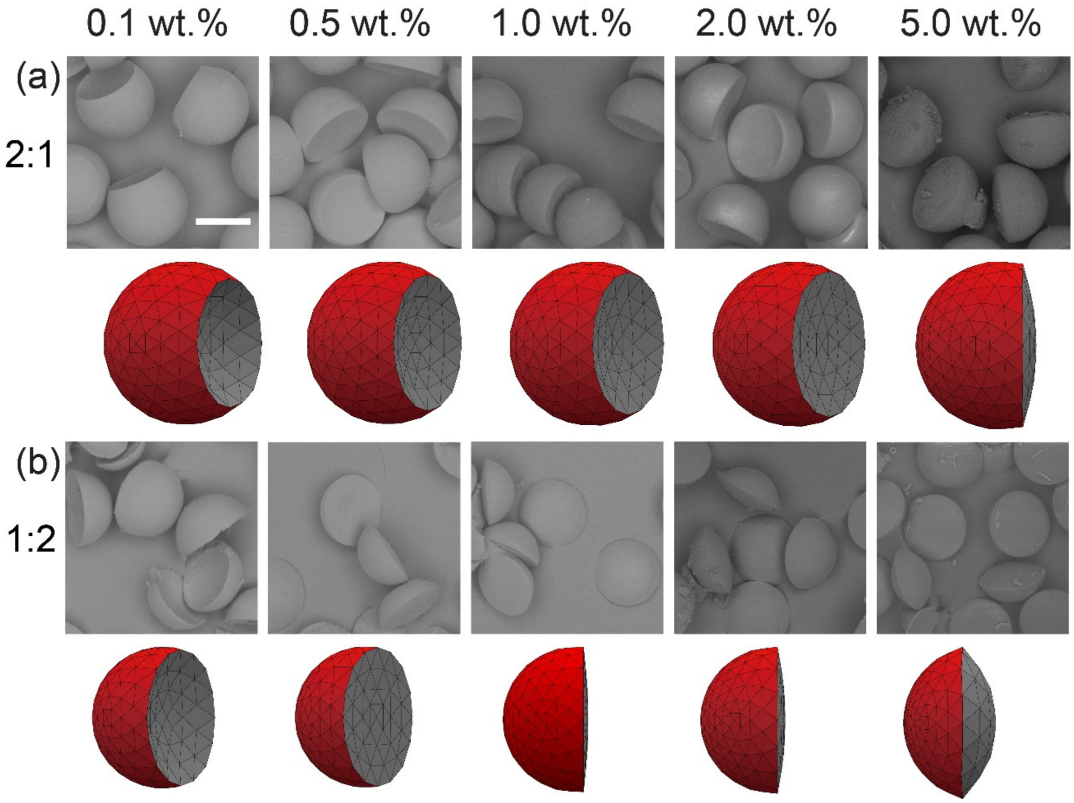

Figure S7. Lens-shaped polymer particles (top) and their Surface Evolver models (bottom) prepared with different surfactant concentrations and flow-rate ratios. (a) Convex-concave and biconvex particles templated from the Janus droplets produced at Q_m/Q_s of 2/1. (b) Convex-concave and biconvex particles template from the Janus droplets produced at Q_m/Q_s of 1/2. The concentration of surfactant in the SO phase ranged from 0.1 to 5.0wt.%. Precursor droplets were produced at $Q_{d,\ total}$ = 0.6 mL h^{-1}, and Q_c = 8.0 mL h^{-1}. Scale bar: 100 μm.
